# Psychological Contract Breach and Outcomes: A Systematic Review of Reviews

**DOI:** 10.3390/ijerph192315527

**Published:** 2022-11-23

**Authors:** Gabriela Topa, Mercedes Aranda-Carmena, Berta De-Maria

**Affiliations:** 1 Faculty of Psychology, UNED—Universidad Nacional de Educación a Distancia, 28040 Madrid, Spain; 2 Universidad Autónoma de Chile, Santiago 7500912, Chile; 3 Faculty of Health Sciences, Department of Psychology, Universidad Rey Juan Carlos, 28933 Madrid, Spain

**Keywords:** psychological contract, psychological contract breach, systematic review, review of reviews

## Abstract

A psychological contract is a set of individual beliefs that a person has about the reciprocal obligations and benefits established in an exchange relationship, such as an employment relationship in an organizational setting. A psychological contract breach is a subjective experience referred to the perception of one of the parties that the other has failed to adequately fulfill its obligations and promises. Breaches have been systematically connected to employees’ attitudes and behaviors that hamper the employment relationship. Despite its apparent clarity, some relevant topics about psychological contract breach, psychological contract fulfillment and the relationships with their consequences still remain unclear. The main objective of this review of reviews is to conduct a review of reviews on psychological contract breaches, considering both systematic reviews and metanalytical papers with the purpose of synthesizing the evidence to date under the psychological contract theory. Using the SPIDER tool, our systematic review of reviews focuses on: (a) Sample; (b) Phenomenon of Interest; (c) Design; (d) Evaluation; and (e) Research type. Finally, only eight systematic reviews and meta-analyses met the inclusion criteria. Of the eight reviews included, seven were meta-analyses while the other was a systematic quantitative review. This study describes the available empirical research on psychological contract breaches and fulfillment and summarizes the meta-analytical evidence on their relationships with attitudinal and behavioral outcomes, as well as the role of potential moderator variables. Due to the methodological caveats of the reviews themselves and of the primary studies they were based on, our conclusions about the impact of psychological contract breaches on outcomes still remain tentative.

## 1. Introduction

Psychological contract theory has its roots in social exchange theory [1] and it has been extensively applied to understanding employment relationships since the seminal works of Rousseau [2,3]. While empirical research increased in the first decades of the XXI century [4], some contradictory findings on its relationships with work-related attitudes and behaviors deserve additional attention. For example, recent counterintuitive findings showed that the relationships between psychological contract breaches and both work engagement and in-role performance were absent, while the relationships with Organizational Citizenship Behaviors (OCBs) were positive, when the behaviors were directed to the individuals, but were negative when they were focused on the organization [5]. This increasingly number of empirical studies promoted the publication of meta-analyses, systematic reviews, and critical revisions, but there is still a debate about the usefulness of psychological contract, the strength of the relationships between psychological contract breaches or fulfillment and outcomes, as well as the potential moderators under which these relationships are manifested.

In the last decade, different comprehensive models of the psychological contract have been proposed [6], but, at the same time, some criticisms arose, such as the psychologization of employment relationships [7]. These critiques pointed out that a psychological contract reduces the analysis to the individual level, highlighting the role of individual differences in explaining work-related outcomes, instead of including the collective factors that affect employment and HRM areas. Given that a systematic review of reviews could help us in revealing trends, beyond the data of a specific empirical synthesis [8], we present a global assessment of the findings of meta-analytical studies and reviews focused on psychological contract breaches and fulfillment. The purpose of this systematic review of reviews is to provide an up-to-date synthesis of the empirical evidence, evaluate the quality of the systematic reviews included, and map the advancements and caveats of the psychological contract theory.

### Conceptualizing Psychological Contracts: Content, Type, Breaches, Fulfillment, and Aftermath

In recent decades, the psychological contract has emerged as a broad theoretical model that contributes to the explanation of complex and changing employment relationships. However, the empirical studies that apply it contain some variable and inconsistent findings, for whose interpretation quantitative syntheses would be useful.

A psychological contract is a set of individual beliefs that the person has about the reciprocal obligations and benefits established in an exchange relationship [9,10,11,12]. Although there is no absolute consensus regarding its definition, most authors point out that a psychological contract focuses on the promises that the parties have exchanged in the constituent phases of said contract, and therefore a resulting balance is required to compare what has been promised and what is actually fulfilled. The content of psychological contracts includes a wide range of exchanges as well as financial compensations, career opportunities, security at work, and work–life balance. Most empirical research has focused on two dimensions of psychological contracts, namely relational and transactional contracts, which can be distinguished as a function of their focus on long-term exchanges of socioemotional resources vs. short-term economic exchanges [13]. These two main types of psychological contracts are rarely pure, and Rousseau developed the concept of balanced psychological contracts as exchanging relationships with a mix of economic and social features [14]. Further explorations about the psychological contracts of volunteers or civil servants highlighted that ideological-related psychological contracts could exist, and inducements and fulfillments could be perceived in a different way by these employees [13]. The evolution of the psychological contract theory has later been enriched by a processual perspective, where the organizational socialization, the influences of veteran coworkers as well as newcomers’ emotions have been integrated in a model psychological contract creation [15].

From these theoretical definitions, the operationalizations of the various components of the model follow two main paths. One of them focuses on the fulfillment of the psychological contract and the other on the breach or perceived violation, exploring in both cases their effects on the attitudes and behavior of the workers. Psychological contract fulfillment is defined as the perception that the reciprocal exchanges between the employee and the organization conform to previous promises and such exchanges are considered the key features of the relationship’s quality. Freese and Schalk [16] proposed the first questionnaire on compliance with the various dimensions of the contract based on previous lists of job expectations and values. This approach, which used attitudinal criteria, such as commitment or identification with the organization, and behavioral criteria, such as absenteeism, originated different ways of operationalizing the measures of the fulfillment of psychological contracts. However, it seems to lose prominence to the other line of research that focused on psychological contract breaches or violation.

A psychological contract breach is a subjective experience that refers to the conception by one of the parties that the other has failed to adequately fulfill their obligations and promises. Associated to the perception of psychological contract breaches, violation refers to an intense and negative emotional reaction of anger and distress and feelings of having been betrayed [14,17]. Following these conceptualizations, a wide range of personal and organizational outcomes of both breaches and violation have been explored. Different studies have explored the consequences of violation on the subsequent psychological contracts of victims that do not abandon the firm [18], as well as proposing a dynamic model where the building blocks of contracts (promises, inducements, contributions, and obligations) change over time and play a different role as a function of the creation, maintenance, renegotiation, or repairing phase of the contracts [19]. Moreover, one of the most recent research developments focused on the comparison of the differential impact of algorithmic management vs. human agents on perception of breaches and later employees’ outcomes [20]. Finally, considering the third parties’ information and the coworkers’ cues regarding violation, Costa and Coyle-Shapiro [21] recently proposed that social influence on an individual’s fulfillment evaluation could affect focal individuals’ psychological contracts. Despite the updates of these theoretical models [6,15,18,21], some relevant topics about psychological contract breaches, psychological contract fulfillment, their measurement, the relationships with their consequences, and the potential moderator variables still remain unclear.

Firstly, in the empirical research, the terms breach and violation of psychological contracts are often used in an equivalent and sometimes confusing way. Rousseau clearly established that a breach is one of the basic forms of the violation of the contract, but later a conceptual distinction was proposed between the cognitive component—the disagreement—and the affective or emotional aspect—the violation—that constitute the experience of the overall perceived breach of the psychological contract. At first glance, it seems clear that psychological contract fulfillment is associated with an increase in desirable results and a decrease in undesirable ones for both the person and the organization, while breach follows an inverse relationship pattern. However, when explored more closely, we find highly variable and even contradictory findings among the meta-analyses and reviews [22,23,24]. Hence, there is not conclusive evidence on the differences or the overlapping between fulfillment and breach, and this lack of clarity justifies the present systematic review of reviews.

Secondly, there is also no consensus as to how to operationalize the various measures, both for fulfillment and for breaking a psychological contract. It is as common to ask people to what extent they believe that their employers comply with the promises made at the beginning of the employment relationship as it is to ask them to indicate whether such levels of compliance are sufficient for them or to request a comparison between what was promised and what was received. Therefore, lacking broadly accepted instruments for assessing psychological contract is a recurrent concern in the reviews [25], showing that this topic also justifies further attention.

Thirdly, a wide range of attitudinal and behavioral outcomes was explored as a consequence of both breaches or fulfillment of psychological contracts, but solid empirical evidence was only found for a limited number of dimensions. Therefore, the most relevant discrepancies among the empirical findings have been examined by reviews and meta-analyses, claiming further synthesis.

Fourthly, due to the fact that employment relationships are usually embedded in particular work contexts, they are affected by different potential moderator variables. Previous meta-analyses have already pointed out that there are a number of variables that can affect the results of empirical studies, especially those more closely related to individual features or specific characteristics of the context of the employment relationship. As Raja, Johns and Ntalianis [26] pointed out, personality traits might affect psychological contracts via the type of contract negotiated, the perception of breach, and the subsequent impact of this perception on work-related attitudes and behaviors. Among the demographic characteristics of employees, age, organizational tenure, and occupational categories seem the most relevant moderators explored by reviews. More recently, national cultural factors and macro-economic trends have been proposed as moderators for the meta-analyses [27,28]. Hence, some clarification is needed about the impact of moderator variables in the relationships between psychological contract breaches and outcomes.

Fifthly, some methodological caveats of empirical research have been consistently highlighted by reviews and meta-analyses. As the quality of the empirical studies was a recurrent concern of the reviews, we evaluate the methodological quality of the included reviews using AMSTAR (Assessment of Multiple Systematic Reviews) [29], a measurement tool for the assessment of systematic reviews, in order to reach more valid conclusions.

Finally, most of the systematic reviews follow the PICO tool, focused on the Population, Intervention, Comparison, and Outcomes as the relevant concepts in the research question. Despite its wide use, the PICO search tool was developed in epidemiology and some of their categories are difficult to apply to correlational primary studies and to qualitative research. Hence, Cooke and colleagues [30] developed a new search strategy tool named SPIDER (Sample, Phenomenon of interest, Design, Evaluation, and Research type). The addition of the *Design* and *Research type* categories was intended to increase the ability of identifying relevant articles. Most of the research about psychological contract breaches and fulfillment conducted in the Work and Organizational Psychology field is correlational, and this fact renders *Comparison group* or *Intervention* irrelevant categories. Therefore, following the SPIDER tool, our systematic review of reviews focuses on: (a) Sample: workers, volunteers, or students currently working in any type of organization; (b) Phenomenon of interest: psychological contract breach or violation, and psychological contract fulfillment; (c) Design: systematic reviews or meta-analyses, including primary studies that reported at least one quantitative assessment of psychological contract breach or violation, or psychological contract fulfillment; (d) Evaluation: perceptions of psychological contract breach or psychological contract fulfillment by self-informed questionnaires; and (e) Research type: quantitative systematic reviews and meta-analytical syntheses of empirical evidence.

To sum up, the main objective of this study is to conduct a review of reviews on psychological contract breach, considering both systematic reviews and metanalytical papers with the purpose of synthesizing the evidence to date under the psychological contract theory. The specific objectives of this review of reviews are the following: to provide an up-to-date synthesis of the empirical evidence, evaluate the quality of the systematic reviews included, and map the advancements and caveats of the psychological contract theory.

## 2. Materials and Methods

### 2.1. Study Design and Literature Search

The following English and non-English language electronic databases were used to retrieve systematic reviews and meta-analyses on Psychological Contracts without any language or time restriction: Web of Science, Scopus, PsychArticles, Psicodoc, PsychINFO, Social Science Citation Index, ERIC, Medline, and Google. Reference lists of review articles and the most relevant journals in the Work and Organizational Psychology area (such as *Journal of Organizational Behavior*, *Human Resource Management Journal*, and *Journal of Vocational Behavior*) were consulted. The keywords were *psychological contract*, *psychological contract breach*, *psychological contract fulfillment, psychological contract violation*, AND *review*. We retrieved a total of 320 references. Because of the small number of reviews retrieved, international experts on the topic were contacted, but unfortunately nobody provided us with other reviews or meta-analyses. This systematic review entailed a critical analysis of articles in the literature and was conducted in accordance with the guidelines of the Preferred Reporting Items for Systematic Reviews and Meta-analyses (PRISMA) statement (see Supplementary Tables S1 and S2). The approval of the Research Ethics Committee was not required because the study was a systematic review. The main search was performed in June 2022 and the updated search in September 2022. A total of 45 published studies were retrieved in full text.

### 2.2. Inclusion and Exclusion Criteria

To be included, reviews had to evaluate empirical studies on psychological contract breaches or fulfillment using organizational samples, such as workers, volunteers, or students currently working in any type of organization, but not students that are not working. The reviews and meta-analyses had to include at least one of the following quantitative measures: psychological contract, psychological contract breach, violation, or psychological contract fulfillment. Only peer-reviewed published literature was included; hence, theses, dissertations, and conference proceedings were excluded. Reviews considered not systematic, such as theoretical papers or *position papers*, book reviews, commentaries, and editorials, were also excluded. The most relevant reason for excluding theoretical and critical reviews was the absence of enough information on the criteria used to include or exclude the primary synthesized papers. No articles were excluded based on language. The inclusion and exclusion criteria are summarized in Table 1.

### 2.3. Study Selection

**Figure 1:**
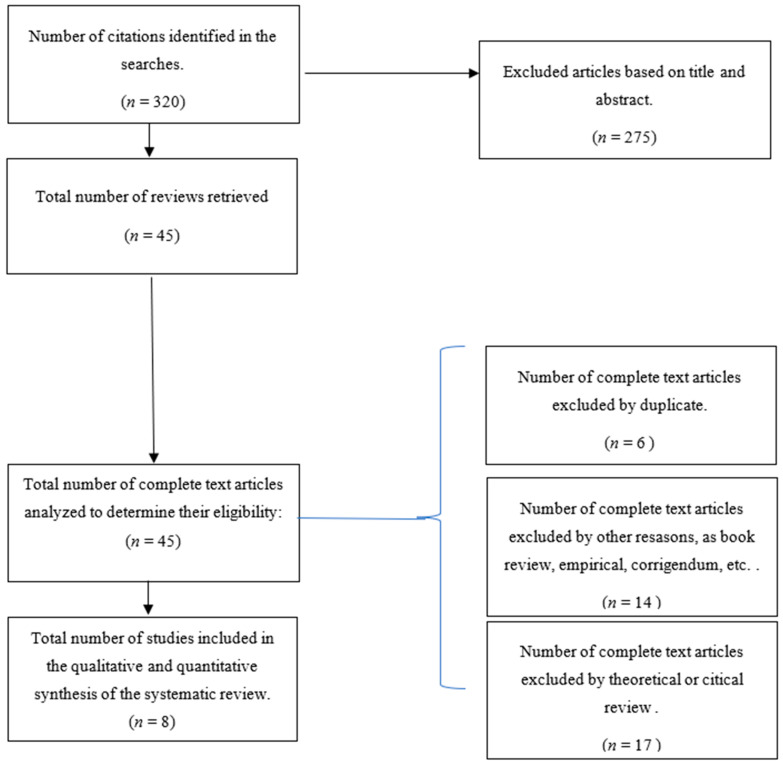
Flow diagram of the information through the different phases.

Based on the above-mentioned criteria of inclusion and exclusion (Table 1), two independent authors screened the list of titles and abstracts retrieved through electronic and manual searches. Any discrepancy was solved by discussion in order to reach a consensus. A total of 45 potentially relevant systematic reviews were retrieved in full text. The further full-text examination of the retrieved papers allowed us to exclude the following: six duplicates, ten book reviews, one empirical study [31], one review focused on other work-related topics [32], one corrigendum article, one review focused on students’ psychological contract [33], and 17 theoretical reviews [6,23,34,35,36,37,38,39,40,41,42,43,44,45,46,47,48]. Finally, only 8 systematic reviews and meta-analyses met the inclusion criteria [22,24,27,28,49,50,51,52]. The included studies are marked in the reference list with an asterisk. Figure 1 is a flow diagram charting the process followed for retrieving the relevant works. The process begun by specifying the number of references extracted from the databases searched. The diagram also specifies the number of documents obtained in the two phases of the process. Firstly, the results for the initial phase (reading of the titles and abstracts) are shown, indicating how non-relevant references (due to either type of document or topic) were removed. Secondly, the diagram specifies the number of references recovered during the final phase (i.e., the reading of the full texts), which allowed us to exclude duplicates, book reviews, corrigendum, empirical studies, reviews focused on students’ psychological contract, and 17 theoretical reviews. The complete list of records retrieved and examined in full text, but that were finally excluded, is in Appendix A.

### 2.4. Data Extraction and Analysis

First, after screening the full text, data collection was conducted in order to identify information on the type of studies included in the reviews, the instruments used, and the outcome or moderator variables analyzed. Other items such as geographical coverage of the review, the time frame of included studies, type of measures, and theoretical background were also identified. Second, we conducted a thorough quantitative analysis of the articles included in this systematic review of reviews using the free software VOSwiever version 1.6.18 (Centerfor Science and Technology Studies, University of Leiden, Leiden, The Netherlands) [53]. It was used to create a network-based map using the titles, keywords, and the abstracts to enrich the quantity of eligible terms. Given that the number of reviews included in this systematic review of reviews was small, with this analysis, we intended to identify the main variables, participants’ features, and studies’ characteristics of the field, and the relationships among them.

### 2.5. Quality of Systematic Reviews

The methodological quality of the systematic reviews was evaluated with AMSTAR, a measurement tool including 11 criteria. The instrument asks reviewers to answer yes, no, cannot answer, or not applicable. The following criteria were considered relevant to our assessment: research question and inclusion criteria established before conducting the review, duplicate study selection, and data extraction by at least two independent researchers, comprehensive literature search, exclusion or inclusion based on the status of publication, or language, publication of a full list of included and excluded studies provided, full information about characteristics of the included studies provided, scientific quality of the included studies assessed, documented, and used in formulating the conclusions, methods used to combine the findings of the studies, the likelihood of publication bias assessed, and conflicts of interest clearly acknowledge. AMSTAR accumulated strong evidence of its reliability and validity. This measurement tool to assess the methodological quality of systematic reviews showed satisfactory inter-observer agreement, reliability, and construct validity in the study conducted by Shea et al. [54]. Items in AMSTAR displayed levels of agreement ranging from moderate to perfect. The global reliability was also adequate.

## 3. Results

The relevant information of the eight titles finally considered for inclusion is summarized in Table 2, Table 3, Table 4, Table 5 and Table 6. Of the eight reviews included, seven were meta-analyses, while the other was a systematic quantitative review. Three reviews were published before 2010, one in 2010, and three in or after 2021.

### 3.1. Description of Included Reviews

The majority of reviews did not provide information about the gender of participants in their primary studies, and those that did offer it provided neither all the descriptive results nor standard deviations [49], perhaps due to the absence of these data in the primary studies that they summarized. Only four reviews included information about the age of primary studies’ participants [22,49,50,51], and only three about participants’ organizational tenure [22,49,50]. In relation to the geographical distribution of the samples participating in the primary studies, only one review did not mention it [24], while the others offered information divided into different categories (e.g., USA vs. North America, including the USA and Canada). The status of publication of the primary studies was mentioned by the majority of the reviews, except two [22,49]. The risk of bias was assessed only by four reviews [22,24,27,50], and all of them used the fail-safe N as indicator. Due to the fact that, when calculating the Orwin’s fail-safe N, it is necessary to specify a value that is considered a “trivial” size, information about the value specified by the researcher is useful for readers, but this information was not provided in any review. Hence, the researcher concludes that publication bias is not a significant problem due to the fact that the fail-safe value was relatively large. Two reviews did not mention the language of the primary studies [50,52]. Most of the reviews included primary studies written in English, with a minor number of studies written in Dutch, Chinese, and other languages. Full information is provided in Table 2 and Table 3.

### 3.2. Quality of the Included Reviews

The methodological quality of the systematic reviews and meta-analyses included was evaluated using AMSTAR, a reliable and valid measurement tool. Two dimensions of the methodological quality of included reviews were assessed: the internal validity of the design (a priori design and clear inclusion criteria; two independent extractors and consensus procedures applied; status of publications explicitly recognized as inclusion criteria or not) and the quality of the information provided by the published review or meta-analysis. The last dimension includes the following features: provision of the complete list of included and excluded studies, information about the features of the primary studies included related to participants, interventions, and outcomes; quality of the included studies assessed and documented; scientific quality of the primary studies used to formulate the conclusions; appropriate methods for combing the finding of studies; likelihood of publication bias informed; and acknowledgement of potential conflicts of interest. The first dimension ranges between 0 and 4, while the second ranges between 0 and 7, with a maximum quality value of 11. The quality of the included meta-analyses ranged from 4.5 to 6.5, and the most frequent failure was the absence of the quality assessment of the primary studies included. The systematic review published by Kutaula et al. [52] had a lower value due to two criteria not being able to be applied (adequacy of the methods used to combine the findings and assessment of the likelihood of publication bias). Full information about the criteria fulfilled by each meta-analysis or systematic review is provided in Table 4.

### 3.3. Antecedent, Outcome, and Moderator Variables

Four meta-analyses included only psychological contract breaches as antecedents, while the other reviews combined this measure with psychological contract fulfillment, psychological contract violation, or assessment of the psychological contract’s content, operationalized as employer’s obligations or relational vs. transactional contract. The outcome evaluations mainly included attitudes such as organizational commitment, job satisfaction, organizational trust, and turnover intention, behavioral outcomes as well as job performance (assessed both globally and separately as in-role performance and OCB), deviant behavior, and actual turnover. The range of potential moderator variables was wide, due to some meta-analyses including the characteristics of the job, the contract, the organization, and studies’ features as moderators [22,49,52], while others were more focused on psychological contracts’ features, employees’ personality and HRM practices, and contextual factors (labor market characteristics or cultural values).

The individual features of participants in the primary studies were included as potential moderator variables. Mainly, the participants’ age was considered as a moderator in four meta-analyses as a categorical variable, as well as organizational tenure, which was included in three meta-analyses. Full information is provided in Table 5.

### 3.4. Strenght of the Relationships between Psychological Contract Breaches and the Outcomes

Considering psychological contract breach as an antecedent, the relationships (average effect sizes) with desired attitudinal outcomes ranged from *r* = −0.45 to *r* = −0.38 for job satisfaction, from *r* = −0.38 to *r* = −0.32 for organizational commitment, and *r* = −0.53 to −0.36 for organizational trust. Related to the desirable behavioral outcomes, the average effect sizes for the relationships between psychological contract breach ranged from *r* = −0.20 to *r* = −0.07 for in-role performance, while it ranged from *r* = −0.31 to *r* = −0.11 for OCB. Undesirable outcomes such as intention to quit, neglect, and turnover were included in a small number of reviews, with their average effect sizes ranging from *r* = 0.36 to *r* = 0.30 for turnover intention. Neglect behavior was only included in two meta-analyses with an average effect size of *r* = 0.21, while actual turnover was included in two meta-analyses, with the average effect size ranging from *r* = 0.13 to *r* = 0.05.

### 3.5. Moderator Variables in the Relationships between Psychological Contract Breach Antecedents and Outcomes

Despite the fact that researchers have hypothesized a wide range of moderators in some meta-analyses (as can be seen in Table 5), few hypotheses have been confirmed. Using categorical moderator variables, Topa and Palaci [50] only confirmed that both the type of employment contract and the occupational category significantly moderate the relationships between psychological contract breach and neglect. Zhao and colleagues [48] confirmed that the content of the psychological contract breach (relational vs. transactional) significantly moderates the relationships with job satisfaction, organizational commitment, turnover intentions, and OCB. Topa, Morales, and Depolo [22] showed that the type of employment contract also moderates the relationships between psychological contract breach and organizational trust.

Using continuous variables, organizational tenure has been shown as the best predictor for attitudinal (job satisfaction and organizational commitment) and behavioral outcomes (OCB and job performance). Specifically, two meta-analyses showed that tenure was the best predictor into the weighted regression analyses predicting job satisfaction, organizational commitment, and OCB, while it fails to predict job performance [22,50]. In the meta-analysis conducted by Bal and colleagues [51], organizational tenure showed a significant predictive power both on job satisfaction and affective commitment, beyond the effect of the employees’ age.

Topa and colleagues’ [22] meta-analysis showed that the quality of studies has a stronger β-value for undesirable, desirable, attitudinal, and behavioral outcomes. Bal and colleagues [49,51] showed a significant moderator effect of the participants’ age on the relationships between psychological contract breach and organizational trust, job satisfaction, and organizational commitment.

Considering contextual economic factors, inflation rate has been a significant moderator of the relationships between psychological contract breach and in-role performance, turnover intention, and actual turnover [27], while the unemployment rate only significantly moderates the relationships between psychological contract breach and in-role performance and turnover intention [27].

The relationships between psychological contract breach and in-role performance are more efficiently moderated by institutional collectivism, power distance, future society, and gender equality practices. The relationships between psychological contract breach and OCB are only more efficiently moderated by institutional collectivism and performance-oriented practices [28]. The relationships between psychological contract breach and intention to quit are only more efficiently moderated by institutional collectivism, future society, and gender equality practices [28]. Finally, the actual turnover–psychological contract breach relationship is only moderated by the future society practices [28].

### 3.6. Clustering Analysis of the Relevant Terms

**Figure 2:**
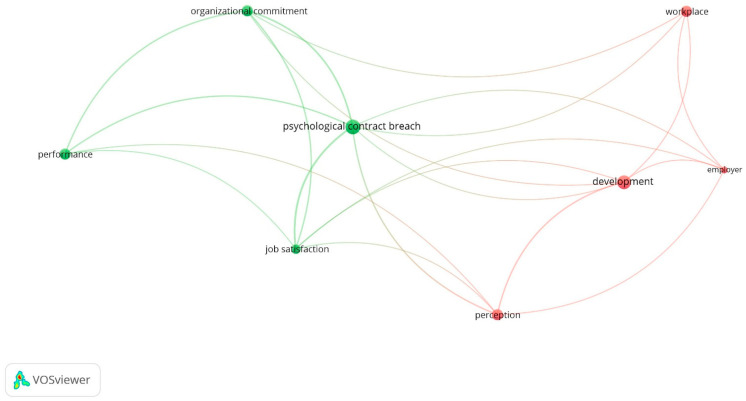
Network map created from the review articles included in this systematic revision using VOSwiever software. Titles, keywords, and abstracts were employed to extract the significant terms.

Psychological contract breach was the central and most frequently used word in the analysis of the relevant terms, as displayed in Figure 2. In the map created from the relevant terms using titles, keywords, and abstracts, breach had the highest value and was clustered with job satisfaction, organizational commitment, and performance (Cluster 1, green), and was directly connected with development, perception, and workplace terms. In cluster 2 (red), development plays a central role and was strongly connected with perception and workplace terms. This network map depicted in Figure 2 reflects a high weight of psychological contract breach as well as its strong connections with attitudinal outcomes and job performance.

## 4. Discussion

This review of reviews on the psychological contract breach and its outcomes had five objectives. First, clarification was needed about the differences or the overlaps between psychological contract fulfillment and breach. Second, the identification of a broadly accepted instrument for assessing psychological contract was also needed. Third, the most relevant discrepancies between primary empirical findings were synthesized. Fourth, clarification about the impact of moderator variables on the relationships between psychological contract breach and outcomes also were needed. Fifth, the quality of the quantitative reviews needed to be evaluated using a measurement tool for the assessment of the methodological quality of the systematic reviews in order to reach more valid conclusions. Based on the above-presented results, we can affirm that some of these objectives were fulfilled, but further research is still needed.

First, the potential differences or overlaps between the measures of psychological contract breach and psychological contract fulfillment still deserve further attention. A separate evaluation of fulfillment versus breach was only reported in one meta-analysis [49]. The other quantitative reviews collapsed both measures into one indicator by reversing the sign of the Pearson’s correlation between fulfillment and outcomes, as Jayaweera and colleagues did [27]. On the one hand, this method combined the data provided by the primary studies and seems theoretically well justified, but precludes us from comparing the strength of the relationships obtained by each one of the evaluation procedures. On the other hand, in the only meta-analysis in which it was included as a predictor, psychological contract fulfillment shows relationships with OCB and organizational commitment, similar to those obtained using psychological contract breach as antecedent, but somewhat smaller. Perhaps, as a consequence of this procedure that collapses fulfillment into breach, our VOSwiever network map, displayed in Figure 2, only included psychological contract breach as a central term.

As some authors recently pointed out [55], reciprocity should be considered as a significant link between psychological contract fulfillment, breach, and violation. Psychological contract fulfillment displays positive reciprocity where employer–employee obligations are respected. Breach highlights the lack of reciprocity and violation that can lead to negative reciprocity in the search of compensation from unfair mistreatment. These relationships between the perceptions of fulfillment, breach, or violation—on the one hand—and outcomes—on the other—could be mediated by attributional processes blaming the firm or its representatives [56], but our findings suggest that fulfillment should not be simply considered as a reversed facet of breach. Moreover, considering that psychological contract fulfillment is an event at work that could exert positive emotions and trigger positive reciprocity, their uncertain role on the relationships between fulfillment and employee’s outcomes should be clarified. However, there is still insufficient information available to comment on the appropriateness of using psychological contract breach or psychological contract fulfillment measures.

Second, the data supported the hypothesized relationships in most of the included meta-analyses and reached a large or medium effect size, except in the case of in-role performance and actual turnover. On the one hand, this finding verifies that the psychological contract breach has very consistent negative consequences on both employees’ attitudes and behaviors. Similarly, the lack of detailed information about the questionnaires used in the primary studies precludes us from reaching any conclusion about the differences between them or their adequacy. Only one review [24] conducted a moderator analysis based on the type of assessment of psychological contract breach (global vs. composite by dimensions). Following their findings, it is possible to conclude that global assessment was less frequent than dimensional assessment, but it renders higher effect size values. Due to the fact that respondents could balance lack of reciprocity in one dimension with its presence in other dimension (e.g., job security vs. payment), when the participants evaluated breaches according to dimensions, they mentioned less discrepancies between promises and fulfillments than when conducting a global assessment of breaches. Given that the standardization of measures remains as a caveat in this area of research [6,25], renewed attempts to develop valid and reliable questionnaires are recommended [57].

Third, this review supports the firm conclusion that psychological contract breaches have a negatively impact on employee outcomes. The effect sizes obtained by the seven meta-analyses seem reasonable and consistent with the theoretical proposals about psychological contract breaches. However, a breach does not reveal an identical level of impact on all outcomes. On the one hand, four attitudinal outcomes were assessed in the majority of the meta-analyses, and three of them reached higher effect sizes: organizational trust, job satisfaction, and organizational commitment. The other attitudinal outcome included in five meta-analyses was turnover intention and showed a positive, but lesser, effect size. Neglect was only assessed in two meta-analyses and showed a lesser effect size. Hence, attitudinal outcomes showed a consistent pattern of effect sizes and low variability was shown between the different meta-analyses. On the other hand, three behavioral outcomes were assessed, in-role performance, OCB, and actual turnover, but the last was only included in three quantitative reviews. Behavioral outcomes only reached low or medium effect sizes, and specifically, the range of the obtained values was higher than that of attitudinal outcomes. Perhaps, actual turnover or decrease in job performance is more related to contextual factors, such as available employment options or financial resources.

To sum up, the impact of psychological contract breaches on attitudes seems to be stronger than the influence exerted on behaviors, but the underlying process remains unclear. In short, one possible explanation is that attitudes appear to be closer to the psychological contract breach than behaviors. As Robinsson, Kraatz, and Rousseau [58] described earlier, a psychological contract breach involves feelings of disloyalty and profounder psychological distress, whereby the victim experiences anger, dislike, a sense of injustice, and unfair damage. Another reason is that the relationship between psychological contract breach and behavior is mediated by behavioral intentions, as the theory of planned behavior suggests. In this sense, these indirect relationships are weaker than the direct ones, which connects psychological contract breach and attitudinal outcome variables. Moreover, another underlying mechanism that could clarify the influence of psychological contract breach on outcomes is provided by the affective events theory [59]. Some authors [60,61] have empirically shown the mediating role of affect in the relationships between different adverse work-related events and employee outcomes. The generation of intense negative psychological states can explain the negative influence of psychological contract breach on employee well-being, operationalized as job satisfaction, organizational trust, and other attitudes. The lower relationship between psychological contract breach and in-role performance can perhaps be attributed to the direct impact that the decline in performance may have on the work situation, an impact that is not as direct in the case of attitudes. While the employer may not perceive a decline in OCBs, they will surely sanction declines in performance, although this process is also moderated by the type of company in which the work activity takes place. Finally, a more recent view on the underlying processes between breach, violation, and subsequent outcomes has been proposed by Tomprou and her colleagues [18] based on self-regulatory processes. When the victim does not have the option of abandoning the employment relationship, they behave in a way that evolves from their coping strategies to four possible outcomes, namely as reactivation, thriving, impairment, or dissolution. Although the mechanisms are complex, interacting with organizational and societal factors, the post-violation model offers a map for investigating the aftermath of violation.

Fourth, some clarification is provided by this review on the impact of moderator variables. On the one hand, organizational tenure as a continuous variable has a positive regression value on all the results. That is, the longer the tenure in the organization, the lesser the impact of psychological contract breach on employee attitudes and behaviors. These results could be due to the fact that a permanent employee may, in the face of psychological contract breaches, weigh the benefits derived from the employment relationship that a temporary worker does not have. It is also possible that those who hold an indefinite contract or are state employees are not willing to relinquish their employment status easily, even if they perceive that some promises have not been adequately fulfilled. This could explain the small effect sizes achieved for the relationship between psychological contract breaches and turnover intention in some meta-analyses. This pattern of relationships seems to be repeated in meta-analyses relating psychological contract breach to job satisfaction. Four meta-analyses [22,40,50,51] included age as continuous variable in order to test its impact on the relationships between psychological contract breaches and outcomes, but their findings were not consistent. While the two meta-analyses carried out by Topa and colleagues [22,50] found only a negligible impact of age in the relationships between psychological contract breach and outcomes, Bal and colleagues [49,51] reached statistically significant effects of age on the relation between psychological contract breach and some outcomes (organizational trust, job satisfaction, and organizational commitment), despite that the percentage of explained variance is very small. Hence, we believe that the literature based on psychological contract breach could gain insights into the processes it investigates if it took into account the idiosyncratic nature and contextual influences that affect psychological contracts in different latitudes, expanding the findings based on employee age, gender, or organizational tenure, as well as the studies of Jayaweera and colleagues [27,28].

Finally, our findings assessing the methodological quality of reviews allow us to affirm that our conclusions about the impact of psychological contract breaches on outcomes remain still tentative. On the one hand, a common criticism of the meta-analyses is related to their procedures of combing empirical findings obtained from primary studies with very different levels of methodological quality [62]. While some authors recommended the inclusion only of those studies with high methodological rigor, others decided to categorize the primary studies based on their methodological quality [63]. It seems probable that the relationships between psychological contract breach and outcomes differ as a function of the rigor of primary studies. In this sense, if those primary sources with lower methodological quality provide effect sizes highly discrepant from the majority of the values, we suppose that this high variability is related to the lower reliability or validity of the primary studies.

Despite its recognized relevance, the quality of the systematic review and the seven meta-analyses included in this review of reviews only reached a mean AMSTAR score that indicates that the quality of the reviews is only moderate. The main weaknesses were the failure of all of the reviews to provide an a priori design, to offer a full list of excluded studies, and to recognize any potential conflicts of interest. Based on the fact that four of the meta-analyses were conducted by the authors of some of the included primary studies, we assume that any conflicts of interest should have been acknowledge. Due to the methodological caveats of the reviews themselves and of the primary studies they are based on, our conclusions about impact of psychological contract breaches on outcomes still remain tentative.

In spite of this caveat, some suggestions for the further development of the research field can be obtained from our findings. First, related to the measurement topic, current proposals of new scales are valuable, but, at the same time, some studies try to conduct experimental research. These designs would allow us to demonstrate causal relationships between psychological contract breaches and their consequences [20,64,65] as well as the impact of orientation programs in the prevention of future breaches [66]. Second, several new ways of research and application of psychological contract breaches have been recently proposed, such as its impact on customer sexual harassment [67], workplace bullying behaviors [68], and expatriates’ psychological contracts [69]. Third, as past research on psychological contract breaches and fulfillment and their outcomes has primary focused on employment relationships, currently, some proposals are trying to apply the psychological contract approach to other not work-related relationships, as the link between students and universities [70] or doctoral supervisory relationships [71]. These attempts of widening the application of the psychological contract to higher education contexts would prove its usefulness as well as provide new research insights. Fourthly, following the directions opened by some of the reviews included in this systematic revision [28], empirical articles continue to explore the potential moderator effect of national cultures on psychological contracts. The exploration of the dimensionality of psychological contracts in Islamic cultures [72] or the role of interpersonal influences as Wasta (Middle East) [73], Guanxi (China), Jeitinho (South America), and Blat/Svyazi (Russia) [74] provide valuable findings on the relevance of cultural features and expand the research field from its initial Western-oriented view. To sum up, the psychological contract seems to be still “alive and kicking” considering its potential usefulness to explain complex relationships in workplaces and other contexts as well as its predictive power on personal wellbeing and valuable organizational outcomes.

## 5. Conclusions

Despite the general findings of this review, it should be noted that the psychological contract appears is a broad and comprehensive theoretical model that can account for an important set of personal and organizational outcomes.

## Figures and Tables

**Table 1:** Inclusion and exclusion criteria.

Categories	Inclusion Criteria	Exclusion Criteria
Sample	Workers, volunteers, or students currently working in any type of organization	Students
Phenomenon of Interest	Psychological contract breach, or violation, and psychological contract fulfillment	Other work-related topics
Design	Systematic reviews or meta-analyses, including primary studies that reported at least one quantitative assessment of psychological contract breach, or violation, or psychological contract fulfillment	Book reviews, primary empirical studies, corrigendum articles, and theoretical and critical reviews
Research type	Quantitative systematic reviews and meta-analytical syntheses of the empirical evidence Studies published in English and other languages Peer-reviewed published literature	Theses, dissertations, and conference proceedings without peer-review process

**Table 2:** Information about the process of retrieving the primary studies, publication search, and other study characteristics.

Study	No. of Registered Studies Retrieved	No. of Primary Studies Screened in Full Text	No. of Primary Studies	No. of Independent Samples	Language of Primary Studies
Topa, G.; Palaci, F. (2004) [50]	96	47	40	47	Not mentioned
Zhao, H. A. O., Wayne, S. J., Glibkowski, B. C., & Bravo, J. (2007) [24]	389	111	51	Not mentioned	English and Chinese
Bal, P. Matthijs; De Lange, Annet H.; Jansen, Paul G. W.; Van Der Velde, Mandy E. G. (2008) [49]	352	Not mentioned	60	62	English and Dutch
Topa, G.; Morales-Domínguez, J.F.; Depolo, M. (2008) [22]	Not mentioned	Not mentioned	38	41	English, Italian, French, and Spanish
Bal, P. Matthijs; de Lange, Annet H.; Jansen, Paul G. W.; van der Velde, Mandy E. G. (2010) [51]	347	157	76	77	English and Dutch
Kutaula, S., Gillani, A., & Budhwar, P. S. (2020) [52]	317	Not mentioned	96	Not mentioned	Not mentioned
Jayaweera, Th.l; Bal, M.; Chudzikowski, K.; de Jong, S. (2020) [27]	2436	172	90	95	English, French, or Dutch
Jayaweera, A. T.; Bal, M.; Chudzikowski, K.; de Jong, S. (2021) [28]	2436	172	90	95	English, French, or Dutch

**Table 3:** Participant characteristics of the included reviews and meta-analyses.

Study	Gender (Percentage of Male) (Mean/S.D.)	Age of Participants (Mean/S.D.)	Organizational Tenure (Mean/S.D.)	Geographical Distribution	Status of Publication	Risk of Bias Assessment
Topa, G.; Palaci, F. (2004) [50]	PCB as predictor: 54.295/14.81; PCF as predictor: 47.95/21.6	PCB as predictor: 36.29/5.12; PCF as predictor: 36.99/6.06	PCB as predictor: 6.91/4.24; PCF as predictor: 6.88/2.91	75% EU, 6.25 % USA, 12.5% Asia, 1% Others	75% published	Fail-safe *k* ranging from 21.8 and 3.8 for PCB/ranging from 108 and 4.2 for PCF
Zhao, H. A. O., Wayne, S. J., Glibkowski, B. C., & Bravo, J. (2007) [24]	Not mentioned	Not mentioned	Not mentioned	Not mentioned	9 unpublished theses and doctoral dissertations; 1 working paper	Fail-safe *k* values ranging from 205 to 3 studies
Bal, P. Matthijs; De Lange, Annet H.; Jansen, Paul G. W.; Van Der Velde, Mandy E. G. (2008) [49]	42%	36.15	7.05 (4.58)	EU (N = 24), USA and NA (N = 26); Asia (N = 8), and 4 Others	Not mentioned	Not mentioned
Topa, G.; Morales-Domínguez, J.F.; Depolo, M. (2008) [22]	Not mentioned	35.28/5.27	7.09/4.26	USA (57.5 %), European, (mainly Italy and Spain, 26 %), and Southeastern Asiatic (15 %)	Not mentioned	Fail-safe *k* values ranging from 41 to 4
Bal, P. Matthijs; de Lange, Annet H.; Jansen, Paul G. W.; van der Velde, Mandy E. G. (2010) [51]	Not mentioned	37.6	Not mentioned	USA and Australia 49%, Europe 36%, and Asia 15%	74% journals, 14% conferences papers, 8% dissertations, and 4% working papers	Not mentioned
Kutaula, S., Gillani, A., & Budhwar, P. S. (2020) [52]	Not mentioned	Not mentioned	Not mentioned	15 countries from Asia	Only published studies	Not mentioned
Jayaweera, Th.l; Bal, M.; Chudzikowski, K.; de Jong, S. (2020) [27]	Not mentioned	Not mentioned	Not mentioned	USA (N = 26), EU (N = 29), Australia (N = 2) and Asia (N = 27)	Only published studies	Fail- safe *k* values ranging from 79 to 2 studies
Jayaweera, A. T.; Bal, M.; Chudzikowski, K.; de Jong, S. (2021) [28]	Not mentioned	Not mentioned	Not mentioned	USA (N = 26), EU (N = 29), Australia (N = 2), and Asia (N = 27)	Only published studies	Not mentioned

PCB: Psychological Contract Breach; PCF: Psychological Contract Fulfillment.

**Table 4:** Quality assessment of the included reviews and meta-analyses using AMSTAR.

Study	Global AMSTAR Score	Internal Validity of the Design ^1^				Quality of the Published Review or Meta-Analyses ^2^						
		*A* Priori Design	Duplicate Study Selection and Data Extraction	Comprehensive Literature Search Performed	Status of Publication	List of Included and Excluded Studies	Primary Studies’ Characteristics	Assessment of Quality	Scientific Quality Used in Conclusions	Methods for Combining Findings	Publication Bias	Conflicts of Interest
Topa, G.; Palaci, F. (2004) [50]	6	0	1	1	1	0	1	0	0	1	1	0
Zhao, H. A. O., Wayne, S. J., Glibkowski, B. C., & Bravo, J. (2007) [24]	6.5	0	1	1	1	0.5 *	1	0	0	1	1	0
Bal, P. Matthijs; De Lange, Annet H.; Jansen, Paul G. W.; Van Der Velde, Mandy E. G. (2008) [49]	5.5	0	1	1	1	0.5 *	1	0	0	1	0	0
Topa, G.; Morales-Domínguez, J.F.; Depolo, M. (2008) [22]	6.5	0	0	1	1	0.5 *	1	1	1	1	1	0
Bal, P. Matthijs; de Lange, Annet H.; Jansen, Paul G. W.; van der Velde, Mandy E. G. (2010) [51]	5.5	0	1	1	1	0.5 *	1	0	0	1	0	0
Kutaula, S., Gillani, A., & Budhwar, P. S. (2020) [52] ^3^	4.5 *	0	1	1	0	0.5 *	1	0	0	N.a.	N.a.	1
Jayaweera, Th.l; Bal, M.; Chudzikowski, K.; de Jong, S. (2020) [27]	6.5	0	1	1	1	0.5 *	1	0	0	1	1	0
Jayaweera, A. T.; Bal, M.; Chudzikowski, K.; de Jong, S. (2021) [28]	6.5	0	1	1	1	0.5 *	1	0	0	1	1	0

Note: ^1^ The first-dimension ranges between 0 and 4. ^2^ The second ranges between 0 and 7, with a maximum quality value of 11. ^3^ The maximum possible quality is 9 instead of 11, due to 2 criteria not being applicable. * 0.5: Only the list of included studies is provided, but not the list of excluded studies. N.a.: not applicable.

**Table 5:** Antecedent, outcome, and moderator variables analyzed by the included reviews and meta-analyses.

Study	Antecedent Evaluations	Outcome Evaluations	Moderator Evaluations	Comparisons between Groups of Studies
Topa, G.; Palaci, F. (2004) [50]	PCB/PCF	Organizational commitment; intention to leave; job satisfaction; organizational trust; neglect; job performance	Type of employment contract; occupational categories; type of firm; design of the study; data collection procedure; publication status; geographical origin of the sample (categorical); participants’ age, gender, and tenure (continuous).	PCB/PCF
Zhao, H. A. O., Wayne, S. J., Glibkowski, B. C., & Bravo, J. (2007) [24]	PCB	Affective (violation and mistrust); attitudinal (job satisfaction, organizational commitment, turnover intentions), and individual effectiveness (actual turnover, ocb, and in-role performance).	Type of breach measure (global vs. composite) and content of the psychological contract breach (transactional vs. relational) (categorical)	Global vs. composite assessment of PCB/ Transactional vs. relational PCB.
Bal, P. Matthijs; De Lange, Annet H.; Jansen, Paul G. W.; Van Der Velde, Mandy E. G. (2008) [49]	PCB	Organizational trust, job satisfaction; organizational commitment	Age (Continuous).	N.a.
Topa, G.; Morales-Domínguez, J.F.; Depolo, M. (2008) [22]	PCB	OCB, organizational commitment, job satisfaction, job performance, organizational trust, intention to leave, neglect.	Type of work contract, occupational categories, type of company, collection data procedure, and origin of the sample (categorical). Participants’ age, gender, tenure and primary studies’ quality (continuous).	Desirable vs. undesirable outcomes; attitudinal vs. behavioral outcomes
Bal, P. Matthijs; de Lange, Annet H.; Jansen, Paul G. W.; van der Velde, Mandy E. G. (2010) [51]	Employer’s Obligations/PCB	Organizational trust, job satisfaction and organizational commitment.	Age and Organizational Tenure, (continuous).	N.a.
Kutaula, S., Gillani, A., & Budhwar, P. S. (2020) [52]	PCB, PCF and Violation/PC Content (relational vs. Transactional)	Job satisfaction, organizational commitment, turnover intention, OCB, in role-performance, deviant behavior, loyalty	Personality traits, HRM practices, POS, P-E fit, leadership, job characteristics, organizational justice, contextual factors as cultural values, Emotional Intelligence and economics trends. Work status, type of company.	N.a.
Jayaweera, Th.l; Bal, M.; Chudzikowski, K.; de Jong, S. (2020) [27]	PCB/PCF	In-role performance; OCB; turnover intentions; and actual turnover	Inflation rate; Unemployment rate. (continuous).	Transactional contracts vs. relational contracts.
Jayaweera, A. T.; Bal, M.; Chudzikowski, K.; de Jong, S. (2021) [28]	PCB	In-role performance; OCB; turnover intentions; and actual turnover	Institutional collectivism; performance-oriented; power distance; future society; uncertainty avoidance; and gender equality practices. (continuous).	Institutional collectivism; performance-oriented; power distance; future society; uncertainty avoidance; and gender equality practices.

PCB: Psychological Contract Breach; PCF: Psychological Contract Fulfillment. N.a.: not applicable.

**Table 6:** Mean effect size for the psychological contract breach–Outcome relationship reported by the included meta-analyses.

Study	Job Satisfaction	Organizational Commitment	Organizational Trust	Turnover Intention	Neglect	In-Role Performance	OCB	Actual Turnover
Topa, G.; Palaci, F. (2004) [50]	−0.43	−0.38	−0.36	0.36	0.20	−0.09	−0.31	n.a.
Zhao, H. A. O., Wayne, S. J., Glibkowski, B. C., & Bravo, J. (2007) [24]	−0.45	−0.32	−0.53	0.34	n.a.	−0.20	−0.11	0.05
Bal, P. Matthijs; De Lange, Annet H.; Jansen, Paul G. W.; Van Der Velde, Mandy E. G. (2008) [49]	−0.43	−0.32	−0.52	n.a.	n.a.	n.a.	n.a.	n.a.
Topa, G.; Morales-Domínguez, J.F.; Depolo, M. (2008) [22]	−0.38	−0.36	−0.46	0.30	0.21	−0.07	−0.29	n.a.
Bal, P. Matthijs; de Lange, Annet H.; Jansen, Paul G. W.; van der Velde, Mandy E. G. (2010)	−0.43	−0.32	−0.52	n.a.	n.a.	n.a.	n.a.	n.a.
Jayaweera, Th.l; Bal, M.; Chudzikowski, K.; de Jong, S. (2020) [27]	n.a.	n.a.	n.a.	0.32	n.a.	−0.21	−0.22	0.13
Jayaweera, A. T.; Bal, M.; Chudzikowski, K.; de Jong, S. (2021) [28]	n.a.	n.a.	n.a.	0.32	n.a.	−0.21	−0.22	0.13

n.a.: not applicable.

## Data Availability

Not applicable.

## Supplementary Materials

The following supporting information can be downloaded at: https://www.mdpi.com/article/10.3390/ijerph192315527/s1, Table S1: PRISMA 2020 Checklist for Systematic Reviews; Table S2: PRISMA 2020 Checklist for Abstract.

## Appendix A

This appendix contains the complete list of excluded articles.

**Table A1:** Full list of excluded studies, all of them retrieved and examined in full text.

Final Decision	Article Title	Author	Journal Title	1	2	3	4	5	Document Type
Excluded/Book review	Book Review: Human Resources, Personnel, and Organizational Behavior: Incentives, Cooperation and Risk Sharing: Economic and Psychological Perspectives on Employment Contracts	Ehrenberg, R.G.	*Industrial & Labor Relations Review*	1989	42	3	473	2	Journal Article
Excluded/Book review	Review of Incentives, Cooperation, and Risk Sharing: Economic and Psychological Perspectives on Employment Contracts.	Nalbantian, H.R.	*Contemporary Psychology*	1989	34	2	200	1	Book Review
Excluded/Book review	Book Review Organizations and the Psychological Contract: Managing People at Work	Guarino, J.	*Employee Responsibilities and Rights Journal*	1998	11	2	155	4	Journal Article
Excluded/Book review	Book reviews. Organizations and the psychological contract.	De Vader, C.L.	*Journal of Occupational & Organizational Psychology*	1999	72	2	253	3	Journal Article
Excluded/Book review	Psychological Contracts in Employment: Cross-National Perspectives (Book Review).	Saari, L.M.	*Personnel Psychology*	2001	54	3	744	3	Review Book
Excluded/Book review	Review of Psychological contracts in employment: Cross-national perspectives.	Saari, L.M.	*Personnel Psychology*	2001	54	3	744	3	Book Review
Excluded/Theoretical review	Flexible employment contracts, the psychological contract and employee outcomes: an analysis and review of the evidence	Guest, D.	*International Journal of Management Reviews*	2004	5	1	1	19	Journal Article
Excluded/Book review	Review of Understanding Psychological Contracts at Work: A Critical Evaluation of Theory and Research.	Macey, W. H.	*Personnel Psychology*	2006	59	3	745	3	Book Review
Excluded/Theoretical review	The psychological contract: A critical review	Cullinane, N.; Dundon, T.	*International Journal of Management Reviews*	2006	8	2	113	17	Journal Article
Excluded/Book review	Review of Understanding psychological contracts at work: A critical evaluation of theory and research.	Brewerton, P.	*Journal of Community & Applied Social Psychology*	2007	17	2	165	3	Book Review
Excluded/Theoretical review	How to measure the psychological contract? A critical criteria-based review of measures.	Freese, Ch.; Schalk, R.	*South African Journal of Psychology*	2008	38	2	269	18	Journal Article
Excluded/Theoretical review	Review of the Literature on the Changing Psychological Contract: Implications on Career Management and Organizations	Lessner, R.; Akdere, M.	*Online Submission*	2008				5	Journal Article
Excluded/Empirical article	Review: Nurses’ work patterns: perceived organizational support and psychological contracts	Blastorah, M.	*Journal of Research in Nursing*	2011	16	6	533	3	Journal Article
Excluded/Book review	Book review: David Guest, Kerstin Isaksson and Hans de Witte (eds), Employment Contracts, Psychological Contracts and Employee Well-being	Burchell, B.; Guest, D.; Isaksson, K.; de Witte, H.	*Work, Employment & Society*	2012	26	3	545	2	Journal Article
Excluded/Book review	Review of Employment contracts, psychological contracts and employee well-being.	Burchell, B.	*Work, Employment and Society*	2012	26	3	545	2	Review-Book
Excluded/Theoretical review	Kavramsal Bağlamı ve Olası Tartışma Alanlarıyla Psikolojik Sözleşme: Bir Gözden Geçirme Çalışması.	Topcu, M.K.; Basim, H.N.	*Journal of Social Sciences*	2015	16	2	83	21	Journal Article
Excluded/Students’ PC	Promises from afar: a model of international student psychological contract in business education.	Bordia, S., Bordia, P., and Restubog, S.L.D.	*Studies in Higher Education*	2015	40	2	212	23	Journal Article
Excluded/Theoretical review	Psychological contract in the light of flexible employment: The review of studies/Kontrakt Psychologiczny W Swietle Elastycznego Zatrudnienia Pracownikow-Przeglad Badan	Żołnierczyk-Zreda, D.	*Medycyna pracy*	2016	67	4	529		Journal Article
Excluded/Theoretical review	Understanding the changing nature of psychological contracts in 21st century organizations: A multiple-foci exchange relationships approach and proposed framework.	Alcover, C.M., Rico, R., Turnley, W. H., and Bolino, M.C.	*Organizational Psychology Review*	2017	7	1	4	32	Journal Article
Excluded/Other topics	The flexpatriate psychological contract: a literature review and future research agenda	Pate, J.; Scullion, H.	*International Journal of Human Resource Management*	2018	29	8	1402	24	Journal Article
Excluded/Theoretical review	Psychological contracts: enhancing understanding of the expatriation experience	O’Donohue, W., Hutchings, K., and Hansen, S.D.	*The International Journal of Human Resource Management*	2018	29	8	1379	22	Journal Article
Excluded/Theoretical review	Promoting perceived insider status of indigenous employees: A review within the psychological contract framework	Caron, J.; Asselin, H.; Beaudoin, J.-M.l.; Muresanu, D.	*Cross Cultural & Strategic Management*	2019	26	4	609	30	Journal Article
Excluded/Theoretical review	When AI meets PC: exploring the implications of workplace social robots and a human–robot psychological contract	Bankins, S., and Formosa, P.	*European Journal of Work and Organizational Psychology*	2019	29	2	215	16	Journal Article
Excluded/Theoretical review	Charting directions for a new research era: Addressing gaps and advancing scholarship in the study of psychological contracts	Bankins, S., Griep, Y., and Hansen, S.D.	*European Journal of Work and Organizational Psychology*	2019	29	2	159	6	Journal Article
Excluded/Theoretical review	Psychological contracts: Back to the future.	Griep, Y., Cooper, C., Robinson, S., Rousseau, D.M., Hansen, S.D., Tomprou, M. & Linde, B.J.	*Handbook of Research on the Psychological Contract atWork. Edward Elgar Publishing. New Horizons in Management series*	2019			397	18	Book Chapter
Excluded/Theoretical review	What do we measure and how do we elicit it? The case for the use of repertory grid technique in multi-party psychological contract research	Sherman, U.P., and Morley, M.J.	*European Journal of Work and Organizational Psychology*	2019	29	2	230	14	Journal Article
Excluded/Theoretical review	Towards a social-cognitive theory of multiple psychological contracts	Knapp, J.R., Diehl, M.R., and Dougan, W.	*European Journal of Work and Organizational Psychology*	2020	29	2	200	14	Journal Article
Excluded/Theoretical review	Impact of psychological contract on employees’ performance: A review.	Sachdeva, G.	*Handbook of research on positive organizational behavior for improved workplace performance.*	2021			201	18	Chapter
Excluded/Corrigendum	The flexpatriate psychological contract: A literature review and future research agenda’: Corrigendum.	Pate, J.; Scullion, H.	*The International Journal of Human Resource Management*	2021	32	3	763	1	Erratum/Correction
Excluded/Theoretical review	The psychological contract and volunteering: A systematic review	Hoye, R.; Kappelides, P.	*Nonprofit Management and Leadership*	2021	31	4	665	27	Journal Article
Excluded/Theoretical review	Conceptualisation of Psychological Contract: Definitions, Typologies and Measurement	Botha, L., and Steyn, R.	*Journal of Social Science Studies*	2021	8	2	1	20	Journal Article

1 Year or publication; 2 Volume number; 3 Issue number; 4 First Page; 5 Page Count.
